# A novel method to derive personalized minimum viable recommendations for type 2 diabetes prevention based on counterfactual explanations

**DOI:** 10.1371/journal.pone.0272825

**Published:** 2022-11-17

**Authors:** Marta Lenatti, Alberto Carlevaro, Aziz Guergachi, Karim Keshavjee, Maurizio Mongelli, Alessia Paglialonga

**Affiliations:** 1 Institute of Electronics, Information Engineering and Telecommunications (IEIIT), National Research Council of Italy (CNR), Rome, Italy; 2 Department of Electrical, Electronics and Telecommunications Engineering and Naval Architecture (DITEN), University of Genoa, Genoa, Italy; 3 Ted Rogers School of Management, Toronto Metropolitan University, Toronto, Canada; 4 Ted Rogers School of Information Technology Management, Toronto Metropolitan University, Toronto, Canada; 5 Department of Mathematics and Statistics, York University, Toronto, Canada; 6 Institute of Health Policy, Management and Evaluation, Dalla Lana School of Public Health, University of Toronto, Toronto, Canada; Sejong University, KOREA, REPUBLIC OF

## Abstract

Despite the growing availability of artificial intelligence models for predicting type 2 diabetes, there is still a lack of personalized approaches to quantify minimum viable changes in biomarkers that may help reduce the individual risk of developing disease. The aim of this article is to develop a new method, based on counterfactual explanations, to generate personalized recommendations to reduce the one-year risk of type 2 diabetes. Ten routinely collected biomarkers extracted from Electronic Medical Records of 2791 patients at low risk and 2791 patients at high risk of type 2 diabetes were analyzed. Two regions characterizing the two classes of patients were estimated using a Support Vector Data Description classifier. Counterfactual explanations (i.e., minimal changes in input features able to change the risk class) were generated for patients at high risk and evaluated using performance metrics (availability, validity, actionability, similarity, and discriminative power) and a qualitative survey administered to seven expert clinicians. Results showed that, on average, the requested minimum viable changes implied a significant reduction of fasting blood sugar, systolic blood pressure, and triglycerides and a significant increase of high-density lipoprotein in patients at risk of diabetes. A significant reduction in body mass index was also recommended in most of the patients at risk, except in females without hypertension. In general, greater changes were recommended in hypertensive patients compared to non-hypertensive ones. The experts were overall satisfied with the proposed approach although in some cases the proposed recommendations were deemed insufficient to reduce the risk in a clinically meaningful way. Future research will focus on a larger set of biomarkers and different comorbidities, also incorporating clinical guidelines whenever possible. Development of additional mathematical and clinical validation approaches will also be of paramount importance.

## Introduction

Diabetes mellitus is a chronic metabolic disorder associated with hyperglycemia, i.e. an increase in blood glucose level that may lead to life-threatening damages to the circulatory and nervous systems. This abnormal rise may be caused by a variety of factors, either related to limited insulin production (Type I diabetes) or to deficiency of cell response to insulin, often referred to as ‘insulin resistance’ (Type II diabetes, T2DM). According to the International Diabetes Federation 2021 Report [1], around 536.6 million people (10.5% of the global population) are affected by diabetes, and the global prevalence of this disease is estimated to increase by 16% by 2045. Diabetes-related health expenditures have dramatically increased in the last few years, reaching an estimated cost of 966 billion dollars in 2021. Particularly, T2DM accounts for 90% of diabetes cases worldwide. Developing prevention strategies to reduce the risk of having T2DM is of paramount importance to avoid the potentially serious complications of this disease. However, early detection can be challenging as patients with T2DM may not develop clearly identifiable symptoms in the initial stages of the disease. As such, data-driven predictive models may help identifying individuals at early risk of developing T2DM.

### Machine learning approaches for T2DM prediction

Several machine learning (ML) models have been proposed to predict the onset of T2DM from clinical measures. ML can help investigate possible hidden patterns in the available data (e.g., clinical information, risk factors, and individual characteristics) to uncover possible mechanisms of disease onset and development [2, 3]. For example, Perveen et al. [4] investigated different ML methods to predict the onset of T2DM using information about individual risk factors of metabolic syndrome including abdominal obesity, cholesterol levels and glucose concentration. Results showed that the highest prediction performance (i.e., sensitivity and F-measure of about 80%) was achieved with a Naïve Bayes classifier coupled with K-medoids under sampling to obtain a balanced dataset. Alghamdi et al. [5] investigated ML ensemble techniques for predicting diabetes by using clinical importance and information Gain Ranking methods to select a subset of features from an initial set of 62 attributes related to cardiorespiratory fitness data. Even in this case the dataset was balanced using sampling techniques (i.e., SMOTE-Synthetic Minority Over-sampling Technique). Different ML models to predict the onset of T2DM using different types of input data (e.g., medical records, lifestyle, socio-demographic factors, family history…) have been explored in recent years, overall achieving satisfactory levels of classification performance (i.e., 80% or higher). In this context, transparent ML techniques like decision trees or logistic regression have been applied to obtain decision rules and feature rankings that highlight the overall importance of different risk factors and clinical features [2]. Moreover, personalized strategies for monitoring the health status of T2DM patients have been proposed. For example, Alfian et al, [6] presented a real-time monitoring system based on Bluetooth Low Energy (BLE)-based sensors and ML models to predict the onset of diabetes and to predict future blood glucose levels. Nevertheless, to the best of our knowledge, there is still a lack of fully interpretable ML approaches able to define personalized preventive recommendations to help patients lower their risk of developing T2DM by targeting individualized changes in biomarkers and modifiable risk factors. The personalized approach is more specific and therefore may be more effective than sample-based ML approaches generating average recommendations across the population.

### Counterfactual explanations

EXplainable Artificial Intelligence (XAI) is a field of artificial intelligence comprising several techniques which are able to provide insights about the inner logic of ML models, ensuring transparency of decision-making processes, therefore fulfilling the “right for explanation” demanded, for example, by the European Union General Data Protection Regulation (GDPR) [7]. XAI techniques either focus on the representation of the overall behavior of the model, providing *global explanations*, or describe the decision-making process of the model for a single specific instance providing *local explanations* [8, 9]. Explainability, transparency and trustworthiness are extremely important requirements, especially when it comes to automated solutions that could make life-critical decisions (e.g., clinical decision support systems, medical robotic systems, or autonomous vehicles) and, as such, XAI techniques have been extensively applied to medical case studies in recent years [10–12]. However, transparency of a model is only one of the several components of trustworthiness and even a fully explainable AI system should be compliant with several other requirements in order to be considered trustworthy [13, 14]. For example, when dealing with medical decision support systems, particular attention must be paid to *Human agency and oversight* as a requirement for Trustworthy AI [13], meaning that AI systems must not limit human autonomy and the decision-making process must guarantee that fundamental rights are respected, without causing any harm.

*Counterfactual explanations* (from now on simply referred to as *counterfactuals*) belong to the family of local XAI techniques. In a binary classification problem, a counterfactual explanation is defined as the set of minimal changes that can be applied to the input features related to a specific instance in order to change its predicted class. In the last few years, different methods for the generation of realistic and feasible counterfactuals able to increase transparency of automated decision making processes have been proposed in the literature [15]. The concept of counterfactual was first adapted to the AI field by Wachter et al. [16]. Since then, various methods for generating counterfactuals have been proposed for tabular data, images, and text and applied to several application domains including, for example, vehicle platooning [17], job hiring platforms [18], and disease prediction [16, 19, 20]. Among the use cases reported by [16], one was related to predicting the probability of developing diabetes by performing a logistic regression. To do so, eight features were extracted from the Pima Indians Diabetes Database [21] and counterfactuals were generated to explain the changes in the input features able to provide a probability of having diabetes of 0.5. The same dataset was also used by White et al. [22] to demonstrate their proposed method, called CLEAR (Counterfactual Local Explanations via Regression), to generate counterfactuals through regression coefficients. Specifically, in [22] counterfactuals are generated for each observation by following a brute force approach where small perturbations are applied to each input feature separately and independently. Then, a linear regressor is trained on a balanced neighborhood around the observations. Finally, the regressor is used to determine the counterfactuals and the accuracy of these regressions with respect to the generated counterfactuals is estimated. By applying the CLEAR method, a perturbation of -0.557 in glucose concentration was identified as the minimal change able to generate a decrease in the probability of having diabetes from 0.69 to less than 0.5. However, all the aforementioned studies aimed at developing general methods for counterfactuals generation and did not specifically address constraints related to diabetes prevention for possible application in clinical practice.

### Rationale and contribution

The aim of this study is the development of a novel method based on counterfactual explanations to produce *personalized* minimum viable modifications of routinely measured biomarkers potentially able to reduce the risk of developing T2DM. As a first step in this direction, we recently introduced and validated a method for building counterfactual explanations starting from non-linear envelopes enclosing the points of each output class, using a Two-Class Support Vector Data Descriptor (TC-SVDD) [23] on data from vehicle platooning collision detection [17]. In the same contribution, we extracted a fully transparent, rule-based description of the identified regions to characterize the two output classes and demonstrated the ability of the proposed counterfactuals generation method to determine changes in output class. In [19], we applied the proposed method to characterize T2DM using an unbalanced set of 1857 subjects (428 diagnosed with T2DM and 1429 without the disease) and biomarkers derived from electronic medical records (EMRs). We demonstrated that the minimal variations in the input features associated with a change in the output class were coherent with the literature related to T2DM. Specifically, diabetic patients were on average associated with higher fasting blood sugar (FBS), higher body mass index (BMI), and lower high-density lipoproteins (HDL), compared to their non-diabetic counterfactuals. The method relied on the definition of two TC-SVDD classification regions named “T2DM” and “No T2DM” and on the generation of a set of counterfactuals that, being by definition at minimum distance, were located near the decision boundary of the “No T2DM” class. However, the method developed in [19] is nor readily applicable to diabetes prevention and risk reduction for the following reasons. First, the boundaries of the two regions are very close to each other and, as a result, the observed changes in biomarkers may not be able to decrease the risk of disease and, as such, may not be translated into practical preventive recommendations. In principle, larger changes may be obtained by using smaller regions to define the “No T2DM” class. For example, by reducing the false negative rate (FNR) of the TC-SVDD classifier, a smaller, more conservative, “No T2DM” region can be obtained and used to characterize patients without the disease that are inherently different from those in the “T2DM” region. Second, in [19], the counterfactuals were assessed only in terms of average differences and no human validation of the observed changes in biomarkers was performed. Last, in [19] we focused on characterization of patients already diagnosed with T2DM, rather than on the investigation of preventive recommendations on individuals at risk of developing T2DM in the future. For a clear identification of actionable counterfactuals able to reduce the risk of developing disease, a different dataset than the one used in [19] is needed.

The main contributions and advancements of the present study compared to previous literature and, particularly, compared to [17, 19] are summarized in the followings:

- selection of a dataset including individual observations before the onset of T2DM to investigate which biomarkers and which change in biomarkers can help reduce the risk of developing T2DM;
- development of a novel methodology for the generation of *actionable* counterfactual explanations from numerical and categorical tabular data by varying only a subset of *controllable* features and constraining *non controllable* features such as age and sex;
- generalization of the TC-SVDD classifier that defines the two regions of the output classes by controlling the FNR to modulate the risk associated with the “low” risk output class and obtain “more conservative” minimal changes towards a lower risk of developing T2DM;
- assessment of the proposed XAI framework through an *ad-hoc* survey delivered to medical experts, in line with the *Human agency and oversight* requirement of trustworthy AI;
- comparison of the newly proposed methodology with two state-of-the-art local XAI techniques.

### Structure of the article

The rest of the article is structured as follows. Section *Materials and Methods* introduces the dataset, the proposed counterfactuals generation process and the approach used for counterfactual analysis, including computational and expert based validation. Section *Results* shows the characterization of counterfactuals generated using both the original TC-SVDD and a modified version based on FNR control. The section also presents the main outcomes of the expert assessment and the comparison with two local XAI techniques. In section *Discussion*, the main findings are examined in the context of available literature and knowledge, highlighting limitations and possible future research. Finally, the last section (*Conclusion*) summarizes the main remarks and outlines directions for further development.

## Materials and methods

### Dataset

The dataset analyzed in this study includes a set of records extracted from the Canadian Primary Care Sentinel Surveillance Network (CPCSSN), a Canadian longitudinal database of EMRs [24]. The protocol was submitted to the Toronto Metropolitan University (formerly Ryerson University) Review Ethics Board (REB 2013–261) and the Board provided a waiver of review as the portion of CPCSSN database used includes de-identified and anonymized records. Specifically, the CPCSSN database here used includes primary care EMR data collected from January 1, 2002 to June 31, 2015 from 1’283’154 patients overall, 90’278 of which diagnosed with diabetes (including T2DM, Type I diabetes, and gestational diabetes). Each patient is associated with several records in the database including, for example, data from medical encounters, laboratory results, results of physical examinations, and medical prescriptions. In this study, in addition to general patient characteristics such as *age* and *gender*, eight biomarkers, routinely measured in primary care, have been considered. We selected the following features based on their relevance to T2DM risk prediction [1, 25, 26] and their large availability in the general population: *fasting blood sugar* (FBS, mmol/L), *body mass index* (BMI, kg/m2), *systolic blood pressure* (sBP, mmHg), *high-density lipoprotein* (HDL, mmol/L), *low-density lipoprotein* (LDL, mmol/L), *triglycerides* (TG, mmol/L), *Total Cholesterol* (Total Chol, mmol/L), and *presence of hypertension*(HTN, {0,1}). The hypertension variable in CPCSSN codifies the presence of essential hypertension, hypertensive heart disease, hypertensive chronic kidney disease, hypertensive heart and kidney disease, and secondary hypertension. In all cases there must be a clear diagnosis of hypertension.

Inclusion criteria for diabetic patients were: age ≥ 30, diagnosis of T2DM, presence of at least one medical encounter up to one year before the onset of T2DM, and presence of at least one value for *each* biomarker in a time window from six years to one year before diagnosis. By applying these criteria, 2791 T2DM patients were selected. Inclusion criteria for non diabetic patients were: age ≥ 30, absence of any kind of diabetes diagnosis, presence of at least two encounters (with a difference of at least one year), and presence of at least one value for *each* biomarker in a time window from six years to one year before the last encounter. In order to obtain a balanced dataset, 2791 non diabetic patients were randomly selected from about 58000 eligible non diabetic patients.

For each patient, a single record was obtained by averaging all the available readings for each biomarker in the observation window. Records related to T2DM patients were labeled as “high risk” (i.e., output class *highT2DM*) whereas records related to patients who did not develop diabetes in the observation window were labeled as “low risk” (i.e., output class (*lowT2DM*). As a result, the final balanced dataset consisted of 5882 records, each including 10 input features and one binary output related to the risk of developing T2DM. The *highT2DM* class included data from 1510 females (age: 57 ± 13 years) and 1281 males (age: 58 ± 11 years), whereas the *lowT2DM* class included data from 1710 females (age: 58 ± 11 years) and 1081 males (age: 60 ± 11 years).

### Counterfactuals generation process

**Fig 1:**
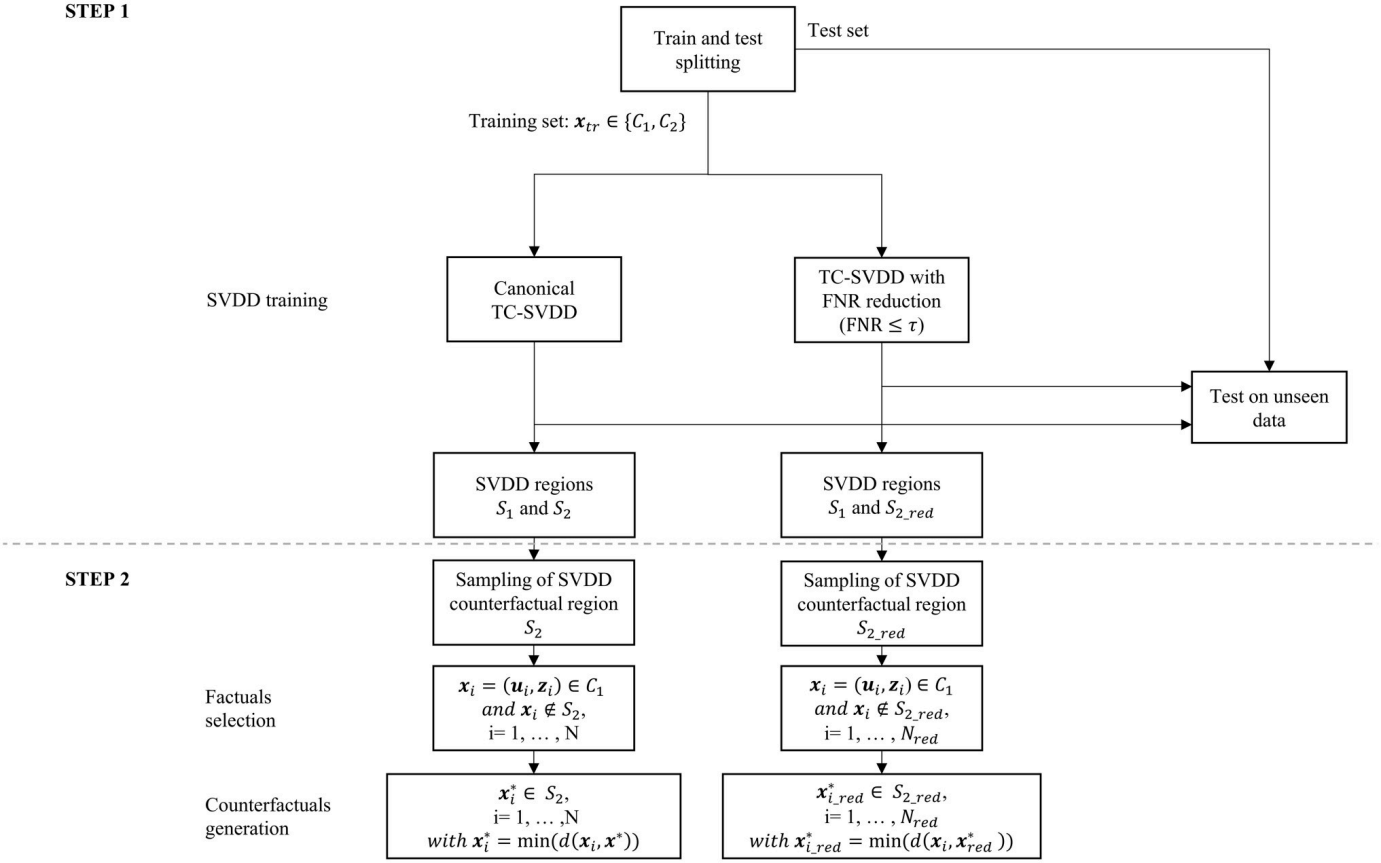
Workflow. Schematic workflow of the counterfactuals generation process.

Fig 1 shows a schematic workflow of the proposed counterfactuals generation method. First, the regions characterizing patients in the two ouput classes are identified (see *Step 1. Classification using* TC-SVDD), then counterfactuals are estimated (see *Step 2. Counterfactuals search algorithm*). The counterfactual generation process code (https://github.com/AlbiCarle/CounterfactualSVDD) was implemented in Matlab R2021a.

#### Step 1. Classification using TC-SVDD

The dataset is divided into training (70%) and test (30%) sets by applying stratification to maintain class balancing in the partitions. Then, a TC-SVDD [23] is trained over training data to learn the decision boundaries of two classification regions, i.e. *S*_1_ (*highT2DM* class) and *S*_2_ (*lowT2DM* class), with *S*_2_ being the target region, where searching for counterfactuals.

More specifically, given a set of data points {(**x**_*i*_, *y*_*i*_)} (where *y*_*i*_ is the label that determines to which class, I or II, the observation belongs) and a binary classification problem, the aim of the TC-SVDD is to find a region (i.e. an hypersphere with center **a** and radius *R*) for each class that encloses as many points of that class as possible, while limiting the volume of the region. This is achieved by solving an optimization problem:

$$\begin{array}{c} \text{min}\;F\left(R_{1},R_{2};a_{1},a_{2}\right)=R_{1}^{2}+R_{2}^{2} \end{array}$$ (1)

under the constraints

$$\begin{array}{c} ∥x_{i}-a_{1}{∥}^{2}\leq R_{1}^{2}\;\forall i\;\text{s.t.}\;x_{i}\;\text{belongs}\;\text{to}\;\text{class}\;\text{I},\;\;\;∥x_{i}-a_{2}{∥}^{2}\leq R_{2}^{2}\;\forall i\;\text{s.t.}\;x_{i}\;\text{belongs}\;\text{to}\;\text{class}\;\text{II}\\ ∥x_{i}-a_{2}{∥}^{2}\geq R_{2}^{2}\;\forall i\;\text{s.t.}\;x_{i}\;\text{belongs}\;\text{to}\;\text{class}\;\text{I},\;\;\;∥x_{i}-a_{1}{∥}^{2}\geq R_{1}^{2}\;\forall i\;\text{s.t.}\;x_{i}\;\text{belongs}\;\text{to}\;\text{class}\;\text{II} \end{array}$$

Since the algorithm tries to maximize the number of correctly classified points by minimizing the volume of the spheres, the TC-SVDD algorithm abstains from making a final decision on a subset of data points, namely the *unclassified* points, i.e., not belonging to either region as they represent data in an overlapping region between the two classes. Although the feature space is not entirely mapped into two classes, the TC-SVDD algorithm generates two closed, dense regions (*S*_1_ and *S*_2_) with high reliability in classifying points belonging to the *highT2DM* and *lowT2DM* class, respectively. As a result, the TC-SVDD yields high positive predictive value (PPV) and negative predictive value (NPV). The TC-SVDD algorithm fits our goal since the search for counterfactuals must be performed in a well-specified closed region to maximize the reliability of the counterfactuals generated. The aforementioned optimization problem can be relaxed and be solved analytically by introducing slack variables and regularization parameters (please refer to [23] for further details). However, it is worth mentioning that the regularization parameters play a relevant role in training the classifier, as they handle the amount of error allowed between classes. The introduction of a radial basis function kernel allows to map the input data points into a high dimensional feature space, to increase flexibility. A set of hyperparameters has been selected for the TC-SVDD training, including the width of the kernel (*σ* = 5) and the regularization terms *C*_1_ = 1, *C*_2_ = 1, *C*_3_ = 1/(*νN*_*highT*2*DM*_), *C*_4_ = 1/(*νN*_*lowT*2*DM*_), where *ν* is equal to 0.05 [23] and *N*_*highT*2*DM*_ and *N*_*lowT*2*DM*_ are the number of training points belonging to the two output classes, respectively. These latter terms establish a trade-off between volume and classification errors of *S*_1_ and *S*_2_, respectively.

It is worth noting that maximization of classification accuracy may not lead to sufficiently reliable counterfactuals in some applications, for example those characterized by fluctuating data or inherent overlap between classes, such as typical disease prediction problems. In these cases, reducing the descriptive regions to characterize a smaller but more stable set of points in the target classes can help increase the reliability of the generated counterfactuals. This could be obtained by implementing a *reliable* version of the TC-SVDD which tries to minimize the number of misclassified points in one or both classes. A possibility is to limit the false positive rate (FPR) and/or the FNR i.e., the conditional probability of a positive prediction given a negative observation and/or the conditional probability of a negative prediction given a positive observation. In our task of disease risk reduction, for example, implementing such an approach may be useful to define a “more conservative” *lowT2DM* region which includes data from patients with presumably better health, i.e. points at a higher distance from points in the opposite class. Let’s consider *lowT2DM* as the negative class and *highT2DM* as the positive class. To define a “more conservative” *lowT2DM* region (*S*_2_*red*_), the FNR can be kept below a given threshold (at the expense of accuracy). In the specific version of the SVDD here used (i.e. *TC* − *SVDD*_*red*_), the center *a* and radius *R* characterizing the shape of the SVDD region *S*_2_ are modified iteratively during the training phase until the FNR reaches a value below a certain threshold *τ* [27, 28]. The threshold *τ* depends on the specific application. In a disease classification and prediction problem based on routinely collected biomarkers, like the one presented in this study, typically there is inherent overlap between the two classes and the distributions of features exhibit high variability. Hence, values of FNR close to zero could lead to an excessively small *S*_2_ region, poorly descriptive of the *lowT2DM* class, thus causing loss of meaningful information regarding healthy patients. In this study, *τ* was set at 8% following a preliminary analysis to establish a trade-off between the percentage of true negative points within the region (e.g., >35% of the training set) and FNR value.

#### Step 2. Counterfactuals search algorithm

Once the two classification regions *S*_1_ and *S*_2_ have been defined, *S*_2_ is discretized with a low-discrepancy method based on the quasi-random Halton sequence [29] to obtain the set of candidate counterfactuals. The use of a low discrepancy method allows to obtain a spatially balanced sampling of the features space and ensures convergence. Indeed, if we assume to sample *L* points starting from the continuous set of points *S*_2_, convergence is guaranteed with a rate of $\text{O}(\frac{1}{L})$, which is faster with respect to the convergence rate ensured by random discretization of points (i.e., $\text{O}(\frac{1}{L^{1/2}})$) [30]. Specifically, *L* was set equal to 10000 in this study. Counterfactuals are generated from real *highT2DM* observations, called *factuals*. Specifically, for each factual **x**_*i*_, the corresponding counterfactual is defined as the point $x_{i}^{*}=x_{i}+\Delta x_{i}$ belonging to the sampled region *S*_2_ (and not belonging to *S*_1_) that lies at minimum distance (i.e., Euclidean distance in our study) from the factual:

$$\begin{array}{c} \underset{\Delta x_{i}\in R^{n}}{\text{min}}d(x_{i},\left(x_{i}+\Delta x_{i}\right))\;\text{subject}\;\text{to}\;{∥\left(x_{i}+\Delta x_{i}\right)-a_{1}∥}^{2}\geq R_{1}^{2}\;\text{and}\;{∥\left(x_{i}+\Delta x_{i}\right)-a_{2}∥}^{2}\leq R_{2}^{2} \end{array}$$ (2)

where **a**_1_ and $R_{1}^{2}$, **a**_2_ and $R_{2}^{2}$ are the centers and the radii of the hyperspheres describing regions *S*_1_ and *S*_2_, respectively.

Indeed, unless for the presence of discontinuity points, counterfactuals will be found, by definition, near the boundary of *S*_2_. As such, if no limit on the FNR is applied, the counterfactuals might be characterized by small differences in features compared to their factuals, therefore they may have potentially limited benefit in terms of reducing the risk of developing disease. Moreover, as the counterfactual would be close to points that characterize patients with high risk of disease, a patient with the biomarkers variation described by the counterfactual might be in an unstable situation, since a small fluctuation in biomarkers would bring the patient back to the high-risk region. A more conservative risk reduction can be obtained by searching inside the reduced region *S*_2_*red*_, characterized by FNR below *τ*, while keeping the definition of counterfactual generated at minimum distance. Moreover, the number of available factuals increases (*N*_*red*_ ≥ *N*) as reducing the volume of region *S*_2_ also reduces the overlap region between *S*_1_ and *S*_2_.

As discussed in [17], the set of input variables **x** can be subdivided in two groups: *controllable* variables **u** = (*u*_1_, …, *u*_*n*_) and *non controllable* variables **z** = (*z*_1_, …, *z*_*m*_). Regarding T2DM, controllable variables can be referred to as biomarkers that can be manipulated such as through medications, lifestyle changes, or medical treatments. Non-controllable variables are instead non-manipulable features such as age, sex, and family history. In this study, age, sex and the presence of hypertension were constrained during counterfactual generation. In particular, the presence of hypertension has been considered as partially non-controllable because hypertension is a chronic disease. Although it can be treated with medications and lifestyle changes, its treatment requires long-term interventions, whereas we are focusing on a narrower window of time. Moreover, the CPCSSN codifies an onset date for the disease, but it doesn’t codify an offset date. For this reason, in the proposed framework, if the *highT2DM* factual presents hypertension, its related counterfactual (*lowT2DM*) will also present hypertension due to the chronic nature of this condition. However, although counterintuitive towards the goal of reducing the risk of disease, the algorithm hypothetically allows a non-hypertensive factual to have an hypertensive counterfactual.

### Counterfactuals analysis

#### Analysis of performance

The performance of the TC-SVDD in terms of ability to enclose the two output classes was assessed using common ML metrics like accuracy, specificity, sensitivity, PPV, NPV, FNR, and FPR. The generated counterfactuals were evaluated according to the following properties [15]:

- *Availability*. The counterfactual $x_{i}^{*}$ is available if it is returned by the search algorithm. Availability can be defined as the ratio of the number of counterfactuals to the total number of factuals.
- *Validity*. The counterfactual $x_{i}^{*}$ should belong to a different class from that of the factual. Hence, validity is defined as the ratio between the number of counterfactuals that have the desired class label (i.e., *lowT2DM*) and the total number of counterfactuals generated.
- *Actionability*. To guarantee feasibility, the counterfactual $x_{i}^{*}$ should never change non controllable features. Actionability is the ratio between the number of constrained features and the total number of non controllable input features.
- *Similarity*. The counterfactual $x_{i}^{*}$ should be close to **x**_*i*_, given a distance function d. The lower the distance, the higher the similarity. Specifically, the distance between **x**_*i*_ and $x_{i}^{*}$ should be lower than a predefined threshold *ϵ*, i.e., $d(x_{i},x_{i}^{*})<\varepsilon$. To assess similarity, we normalized data between 0 and 1. Then, we calculated the Euclidean distance between each counterfactual and the corresponding factual and we computed the ratio between the distance and the maximum theoretical distance in the standardized feature space (i.e., $\sqrt{n_{features}}$). The distance distribution observed over the factual-counterfactual pairs was summarized in terms of average and 95% confidence interval (C.I.).
- *Discriminative Power*. Despite being close to **x**_*i*_ based on the desired property of similarity, the counterfactual $x_{i}^{*}$ should be distinguishable from the points of the class to which **x**_*i*_ belongs. Discriminative Power was assessed by evaluating the accuracy of a k-Nearest Neighbor (KNN) classifier (k = 5) trained on a dataset including the counterfactuals and the real *highT2DM* data points. 5-Fold cross-validation was used and the average accuracy on the test set was estimated. Discriminative power was also investigated from a subjective point of view through specific questions incorporated in an Expert survey (see subsection *Expert assessment*).

#### Characterization of counterfactuals

Four different, non overlapping groups of patients have been considered in the analysis of the generated counterfactuals: females with hypertension (*F_HTN*), females without hypertension (*F_noHTN*), males with hypertension (*M_HTN*), males without hypertension (*M_noHTN*). Females and males were considered separately because of the presence of well known gender-related differences in biomarkers, for example BMI and cardiovascular risk factors (e.g., HDL, LDL) [31]. Hypertensive and non-hypertensive patients were considered separately to assess whether the presence of this comorbidity may influence the changes determined by counterfactuals towards a reduced risk of developing T2DM. In each group of patients, the changes for each controllable biomarker **u**_*i*_ have been computed as follows:

$$\begin{array}{c} \Delta u_{i}=u_{i}^{*}-u_{i}=u_{i,lowT2DM}-u_{i,highT2DM} \end{array}$$ (3)

The Lilliefors test was performed to check for normality of the distributions of the changes for each biomarker, in the four groups. If the variables were not normally distributed, non-parametric tests were used. Specifically, the paired samples Wilcoxon signed-rank test was performed to test the presence of significant differences between factuals and counterfactuals for each biomarker and for each group. The Wilcoxon rank-sum test was applied to assess possible differences in the observed changes for each biomarker between different groups of patients. A significance level *alpha* = 0.05 was considered in this study. Bonferroni correction was applied to correct for multiple comparisons. Statistical analysis was performed in Python version 3.7.

#### Expert assessment

Changes in input features suggested by the counterfactuals may be validated by using domain-specific approaches. In some cases, the observed phenomenon may be simulated (see for example [17]) and/or validated with respect to a known or computationally estimated gold standard. In other cases, for example in disease prediction and prevention applications, validation of model predictions against computational simulations is, in general, not possible. A possible way to analyse the quality of the proposed approach is to perform an application-grounded evaluation by asking domain experts (i.e., clinicians) to assess the perceived usefulness and effectiveness of the changes in biomarkers suggested by the counterfactuals, for the purpose of T2DM risk reduction [32].

To perform a preliminary evaluation of the proposed approach, a group of 7 medical experts based in Canada was asked to fill out a survey. All the respondents participated to this phase of the study on a voluntary basis. All data were collected anonymously. The experts were blinded to any details on the proposed method to limit possible bias.

The survey consisted of four sections and is reported in full detail in the supplementary material (S1 File). The first section included general questions about the experts’ clinical background, and their opinion on AI. The second section (*risk evaluation*) included five examples of patients with related input features: 3 real *highT2DM* patients, 1 real *lowT2DM* patient, and 1 *lowT2DM* patient representing a candidate counterfactual in *S*_2_*red*_. The task was to assess the risk the patient had of developing T2DM in 1 year and to provide a confidence level associated with that assessment. Both patient risk and confidence level were selected from a marked five-item scale. The third section (*counterfactuals evaluation*) included four examples of patients with high risk of developing T2DM (i.e., factuals) and the related changes in biomarkers proposed by the algorithm based on *TC* − *SVDD*_*red*_ in order to reduce the risk (i.e., counterfactuals). Each expert was asked to specify the level of agreement with the target values proposed by the algorithm on a marked five-item scale. In the final section, each expert was asked to provide overall feedback on the proposed methodology (e.g., quality, usefulness). The survey was administered online using Microsoft Forms.

#### Comparison with state-of-the-art techniques

We compared our method with two different techniques, a commonly used XAI technique (SHapley Additive exPlanations-SHAP [33]) and a different method for the generation of counterfactuals (Diverse Counterfactual Explanations-DiCE [34]).

SHAP [33] is a local XAI technique derived from game theory that quantifies the marginal contribution that each single feature (i.e., a single player) brings to the model’s outcome (i.e., the game) for a given instance *X* in the sample. SHAP values can be visualized in different ways. For example, the waterfall plot allows to explain why a specific record receives a certain prediction. The plot sums up the positive or negative contribution of each input feature to get the model’s output probability *f*(*X*), starting from a baseline expected value *E*[*f*(*X*)] that is the model output when no features are present, i.e. the initial proportion of classification (0.5 in our balanced dataset). The summary plot instead combines single explanations to get a global insight of the model reasoning in terms of feature relevance and feature effects on the prediction.

Different methods for the generation of counterfactuals have been proposed in the literature [15], however no common benchmark models have been established yet. The DiCE technique [34] was used in this study because it has similar properties to the method here proposed. Specifically, DiCE is defined for tabular datasets, it can handle categorical features, and it allows to define controllable and non controllable features, in line with the desired property of actionability. Counterfactuals were generated using a Random Forest classifier and the model-agnostic implementation of DiCE with independent random sampling of features [35]. The total number of counterfactuals for each factual was set to 1 and the desired class of the counterfactuals was set to “lowT2DM”. To allow for a fair comparison with our method, the same set of factuals (i.e., real highT2DM points) was considered and the same counterfactuals properties were evaluated.

## Results

### Analysis of performance

A set of factuals and related counterfactuals was generated following the process described in Fig 1. Table 1 shows the classification performance of the canonical TC-SVDD on both training and test sets. From an initial set of 734 factuals, 731 counterfactuals were generated (availability = 99.6%; F_HTN: 154, F_noHTN: 237, M_HTN: 134, M_noHTN: 206). All the counterfactuals belonged to the desired class (validity = 100%). The set of counterfactual showed a high discriminative power characterized by an average accuracy of 94% of the KNN classifier. The average distance between factual-counterfactual pairs is 13% of the maximum theoretical distance in the standardized feature space (C.I.: 4%-21%), suggesting high similarity.

**Table 1:** Classification performance of the TC-SVDD.

	Accuracy	Specificity	Sensitivity	PPV	NPV	FNR	FPR
Training	69%	91%	48%	95%	73%	34%	2%
Test	59%	76%	41%	86%	68%	36%	7%

PPV = Positive Predictive Value; NPV = Negative Predictive Value; FNR = False Negative Rate; FPR = False Positive Rate.

*TC* − *SVDD*_*red*_ generated a region *S*_2_*red*_ that included 750 out of 1954 real *lowT2DM* patients in the training set and 260 out of 837 in the test set, providing a precision of 71% and a negative predictive value (NPV) of 83% on both the training set and the test set. Using *TC* − *SVDD*_*red*_, the number of admissible factuals and, in turn, the number of generated counterfactuals increased because the number of *highT2DM* patients outside the *S*_2_*red*_ region increased compared to those outside the *S*_2_. As a result, a total of 882 counterfactuals was generated starting from 1361 factuals (availability = 65%; F_HTN: 104, F_noHTN: 358, M_HTN: 95, M_noHTN: 325). As observed with the canonical TC-SVDD, all the counterfactuals obtained using *TC* − *SVDD*_*red*_ belonged to the desired class (validity = 100%). As expected, the set of counterfactuals obtained using *TC* − *SVDD*_*red*_ showed a higher discriminative power than that obtained using the canonical TC-SVDD (average accuracy of 97% with the KNN classifier). The average distance between factual-counterfactual pairs is 12% of the maximum theoretical distance in the standardized feature space (C.I.: 4%-21%), suggesting high similarity.

### Characterization of counterfactuals

**Fig 2:**
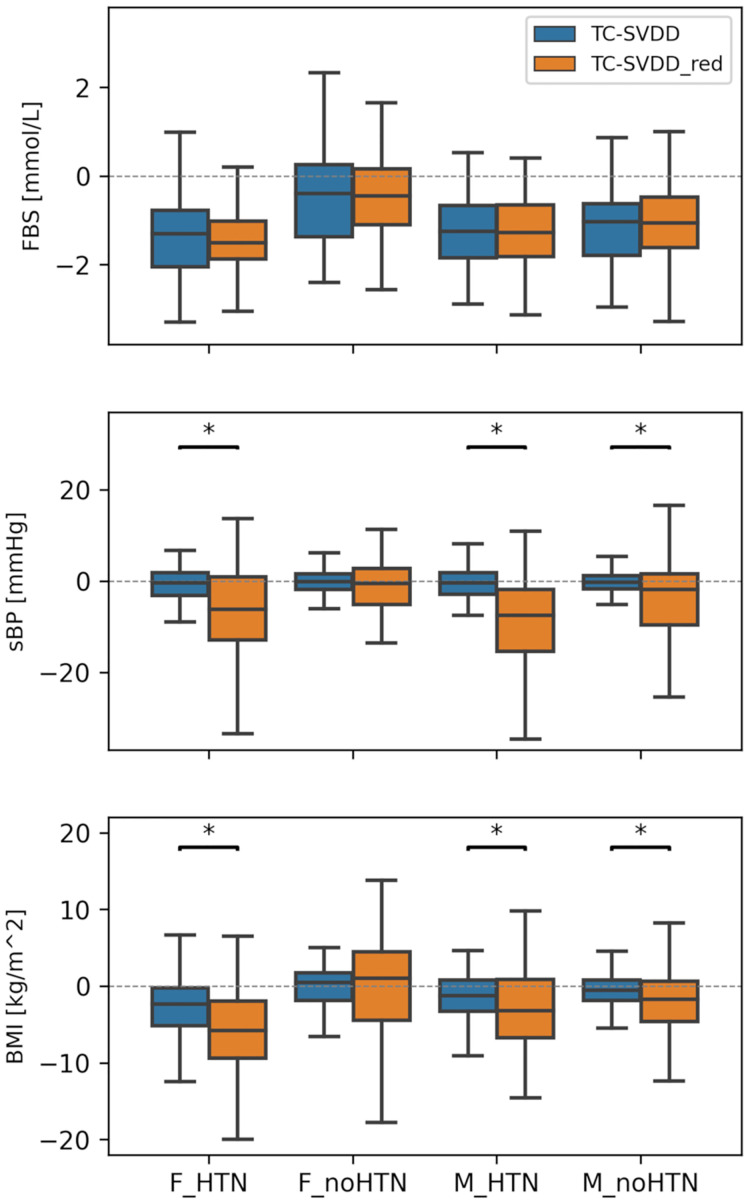
Comparison between Canonical TC-SVDD and TC-SVDD with FNR reduction. Comparison between the changes in FBS, sBP, and BMI derived from counterfactuals generated from a canonical TC-SVDD and TC-SVDD with FNR reductionB(*TC* − *SVDD*_*red*_) in the four groups of subjects: F_HTN, F_noHTN, M_HTN, M_noHTN.

To analyze the differences between the two versions of the TC-SVDD, the changes in biomarkers Δ*u*_*i*_ derived from *S*_2_ and *S*_2_*red*_ have been analyzed. To allow for a fair comparison, only counterfactuals derived from common factuals (i.e., 475) have been considered. As an example, Fig 2 shows the distributions of the changes in FBS, BMI and sBP obtained with a canonical TC-SVDD and *TC* − *SVDD*_*red*_, in the four groups of subjects. Statistically significant differences in median changes in sBP and BMI between the two different TC-SVDD classifiers are observed in hypertensive patients (both F_HTN and M_HTN) and in males without hypertension (sBP—F_HTN: p = 0.006; M_HTN: p <0.001; M_noHTN: p = 0.009; BMI—F_HTN: p = 0.009; M_HTN: p = 0.022; M_noHTN: p = 0.019). Statistically significant differences in median changes between TC-SVDD and *TC* − *SVDD*_*red*_, for the same groups of subjects, have been also found in terms of HDL and Total Cholesterol, whereas no significant changes have been found in terms of FBS, LDL and TG.

**Fig 3:**
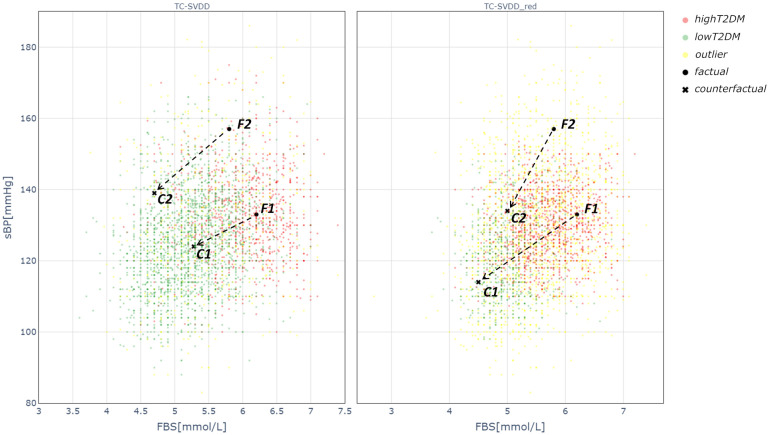
Classification regions obtained with Canonical TC-SVDD and TC-SVDD with FNR reduction. Visualization of classification regions obtained with Canonical TC-SVDD and TC-SVDD with FNR reduction (*TC* − *SVDD*_*red*_) in the plane FBS-sBP with two examples of factuals (black circle markers) and related counterfactuals (black cross markers).

Fig 3 depicts the SVDD representation of the training set points in the FBS-sBP plane, together with two examples of factual-counterfactual pairs. The area of the *lowT2DM* region identified by *TC* − *SVDD*_*red*_ is sensibly reduced with respect to the area identified by the canonical TC-SVDD. As a result, the number of unclassified points increases and the generated counterfactuals are different. For example, considering the factual F1 (FBS = 6.2 mmol/L, sBP = 133 mmHg) the counterfactual generated by *TC* − *SVDD*_*red*_ is associated with lower target values than those generated by the TC-SVDD (i.e., FBS = 4.5 mmol/L, sBP = 114 mmHg vs FBS = 5.3 mmol/L, sBP = 124 mmHg).

The relationship between reduction in sBP and FBS and potential reduction of risk also proves valid when generalizing from individual cases to the entire population since statistically significant differences in median changes of FBS (F_HTN, F_noHTN, M_HTN, M_noHTN: p <0.001) and sBP (F_HTN, F_noHTN, M_HTN, M_noHTN: p <0.001) were found between factuals and counterfactuals for each group of patients, when the *TC* − *SVDD*_*red*_ was applied. Similarly, statistically significant differences in median changes of HDL (F_HTN, F_noHTN, M_HTN, M_noHTN: p <0.001) and TG (F_HTN, F_noHTN, M_noHTN: p <0.001; M_HTN: p = 0.004) were observed for all the four groups, whereas observed differences in median changes of BMI were significant for F_HTN, M_HTN, and M_noHTN (p <0.001), differences in LDL were significant for F_HTN (p = 0.004) and F_noHTN (p <0.001) only, and differences in Total Cholesterol were significant for M_HTN (p = 0.003) and M_noHTN (p = 0.009) only.

Table 2 shows medians and 25th-75th percentile range of the changes (Eq (3)) for each controllable biomarker in the four different groups of patients. Concerning females, the changes in 4 out of 7 controllable biomarkers were significantly different between hypertensive and non-hypertensive patients (FBS, sBP, BMI, TG: p <0.001). Concerning males, 2 out of 7 controllable biomarkers were significantly different between hypertensive and non-hypertensive patients (FBS, sBP: p <0.001).

**Table 2:** Change in biomarkers derived from the counterfactuals generated by *TC* − *SVDD*_*red*_, in four different group of patients: Median (25th percentile; 75th percentile).

	FBS [*mmol*/*L*]	sBP [*mmHg*]	BMI [*kg*/*m*^2^]	HDL [*mmol*/*L*]	LDL [*mmol*/*L*]	TG [*mmol*/*L*]	Total Chol [*mmol*/*L*]
*F_HTN*	-1.25 (-1.74; -0.51)	-4.90 (-15.06; 0.86)	-5.26 (-9.29; 0.48)	0.41 (-0.22; 0.85)	-0.24 (-0.81; 0.31)	-0.49 (-1.10; 0.28)	-0.39 (-1.17; 0.6)
*F_noHTN*	-0.50 (-1.05; 0.13)	-1.19 (-5.41; 2.82)	0.05 (-3.64; 3.08)	0.22 (-0.34; 0.77)	-0.26 (-1.08; 0.57)	0.14 (-0.43; 0.81)	0.04 (-0.90; 0.68)
*M_HTN*	-1.31 (-1.92; -0.70)	-7.5 (-15.59; 0.08)	-2.20 (-7.05; 1.46)	0.68 (0.09; 1.17)	0.10 (-0.79; 0.77)	-0.24 (-0.83; 0.31)	0.52 (-0.43; 1.24)
*M_noHTN*	-0.80 (-1.41; -0.21)	-2.43 (-9.56; 1.37)	-1.98 (-5.12; 1.35)	0.76 (0.17; 1.26)	0.06 (-0.75; 0.75)	-0.23 (-0.91; 0.35)	0.13 (-0.58; 1.07)

### Expert assessment

The group of respondents included 7 clinicians with a diverse range of professional backgrounds: primary care (2), endocrinology, surgery, health informatics, emergency medicine, and dermatology. The survey took on average 29 minutes to complete.

As concerns the first section, all the surveyed experts reported some degree of experience with AI (2 minimal, 2 basic, 3 adequate). The impact AI will have on medical decisions in the coming years was rated as moderate or higher (2 moderate, 3 major, 2 important). Experts had varying opinions on whether an AI-based medical decision support system capable of providing quantitative explanations (through XAI) could be considered fully trustable. Specifically, 1 expert strongly disagreed, 3 were neutral and 3 moderately agreed.

The *risk evaluation* section dealt with the assessment of the patient risk, given a set of biomarkers. Two examples of questions are shown in Table 3. The patient described in EX1 is a female individual, 84 years old, whose FBS is above the clinical prediabetes threshold (i.e., 5.6 mmol/L [1]). The BMI value is in the overweight range (i.e., 25≤ BMI <30). This patient will actually develop diabetes in a year. In this first example, 4 experts rated the patient as having a minor risk, 2 as moderate risk, and 1 as major risk. The majority of the experts claimed to be confident about their evaluation (1 Somewhat confident; 4 Fairly confident). The values reported in EX2 are related to a 56-year-old male patient whose FBS is below the prediabetes threshold. All the other biomarkers are quite similar to those of EX1. This subject will not develop diabetes in a year. In this second example, 1 expert rated the patient as no risk, 3 as minor risk, and 3 as moderate risk.

**Table 3:** Risk evaluation: Examples of subjects at high (EX1) and low risk (EX2) of developing T2DM.

	Gender	Age	FBS [*mmol*/*L*]	BMI [*kg*/*m*^2^]	sBP [*mmHg*]	LDL [*mmol*/*L*]	HDL [*mmol*/*L*]	TG [*mmol*/*L*]	Total Chol [*mmol*/*L*]	HTN
*EX*1: *highT*2*DM*	Female	84	6	27	124	2.6	2.1	1.3	4.7	No
*EX*2: *lowT*2*DM*	Male	56	4.7	27.4	126.5	3.2	1.1	0.9	4.8	No

The *counterfactuals evaluation* section of the survey dealt with the assessment of some examples of *highT2DM* patients (factuals) and the corresponding target changes in biomarkers proposed by the algorithm in order to reduce the risk. Two examples of this kind of questions are shown in Table 4 where two factuals (F1 and F2) and their counterfactuals (C1 and C2) are reported. These factual-counterfactual pairs are the same shown in Fig 3 in the FBS-sBP plane. F1 represents a 63-year-old female patient with hypertension, with FBS above the prediabetes threshold, and slightly elevated BMI (i.e., overweight class). LDL is near the desired range (i.e., optimal if LDL <2.6 mmol/L), HDL is acceptable (i.e., optimal if HDL >1.3 mmol/L in women), TG and Total Cholesterol are in the desired range (i.e., TG <1.7 mmol/L and Total Cholesterol <5.18 mmol/L) according to general guidelines [36]. The algorithm proposes to lower the risk of developing T2DM by targeting the values in C1, namely by reducing FBS, BMI, sBP, TG and Total Cholesterol by keeping the LDL and TG levels almost constant. All the experts agreed that the proposed target values are reasonable to obtain a risk reduction when focusing on T2DM (i.e., 5 Moderately agree; 2 Strongly agree).

**Table 4:** Counterfactuals evaluation: Examples of subjects at high risk of developing T2DM (factuals, F1 and F2) and corresponding counterfactuals (C1 and C2).

	Gender	Age	FBS [*mmol*/*L*]	BMI [*kg*/*m*^2^]	sBP [*mmHg*]	LDL [*mmol*/*L*]	HDL [*mmol*/*L*]	TG [*mmol*/*L*]	Total Chol [*mmol*/*L*]	HTN
*F*1: *highT*2*DM*	Female	63	6.2	28.7	133	3.1	1.1	1.5	4.9	Yes
*C*1: *lowT*2*DM*			4.5	25	114	3.0	0.8	0.4	3.8	
*F*2: *highT*2*DM*	Male	55	5.8	44.1	157	3.0	1.2	2.3	5.9	Yes
*C*2: *lowT*2*DM*			5	40	134	3.0	1.2	2.0	6.2	

F2 represents a 55-year-old male patient living with hypertension, with FBS slightly above the prediabetes threshold and very high BMI (i.e., in the severe obesity range). LDL is near optimal, HDL is optimal (i.e., optimal if HDL >1.0 mmol/L in men), TG and Total Cholesterol are above the desired range. The algorithm proposes to lower the risk of developing T2DM by targeting the values in C2, namely by reducing FBS, BMI, sBP and TG while keeping the other values almost constant. In this case, experts expressed different opinions about the proposed risk reduction strategy (i.e., 3 Moderately disagree; 2 Moderately agree; 2 Strongly agree).

After counterfactuals evaluation, experts were asked to specify which biomarkers were the the most relevant for their assessments (multiple responses were permitted). The following biomarkers were identified as relevant, ordered based on the number of experts who selected them: BMI (7/7), FBS (5/7), Diagnosis of hypertension (3/7), HDL and TG (2/7), sBP and LDL (1/7), Total Cholesterol (0/7).

Expert responses regarding relevant features are largely aligned with the feature ranking obtained by applying the rule based description of the TC-SVDD (following [27]). Specifically, BMI, sBP, FBS, HDL, and diagnosis of Hypertension were the most relevant, whereas TG, Total Cholesterol, LDL were the least relevant.

### Comparison with state-of-the-art techniques

**Fig 4:**
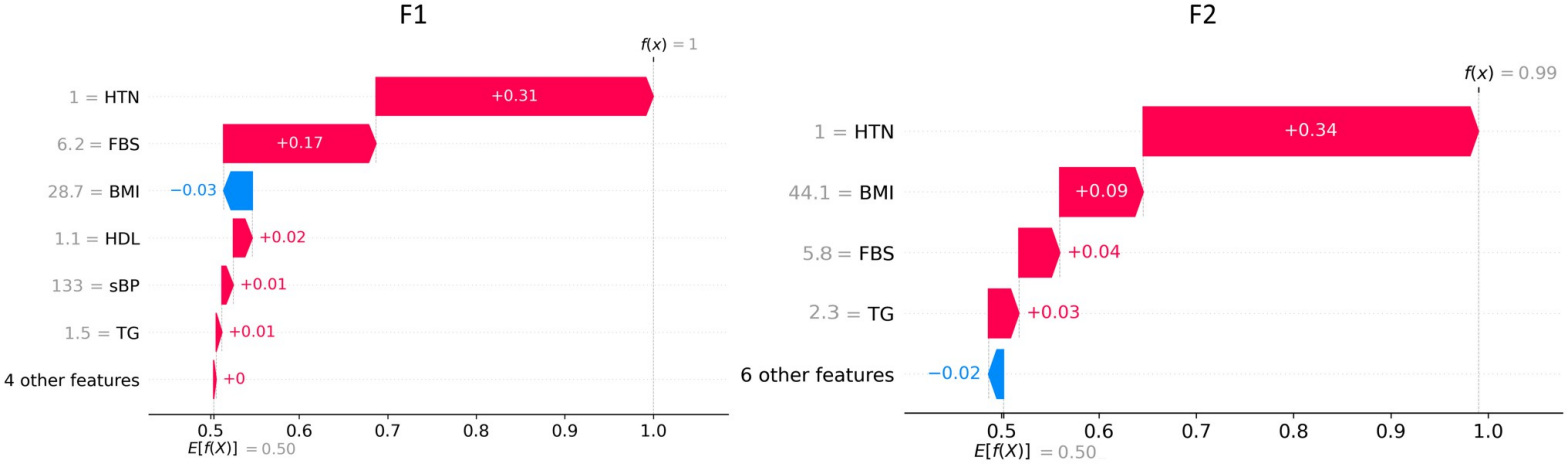
SHAP waterfall plots for individual predictions. Waterfall visualization of SHAP values related to factuals F1 (left panel) and F2 (right panel). Red bar: positive contribution; blue bar: negative contribution. *E*[*f*(*X*)]: baseline expected output; *f*(*X*): output predicted by the model. Features are ordered by importance.

**Fig 5:**
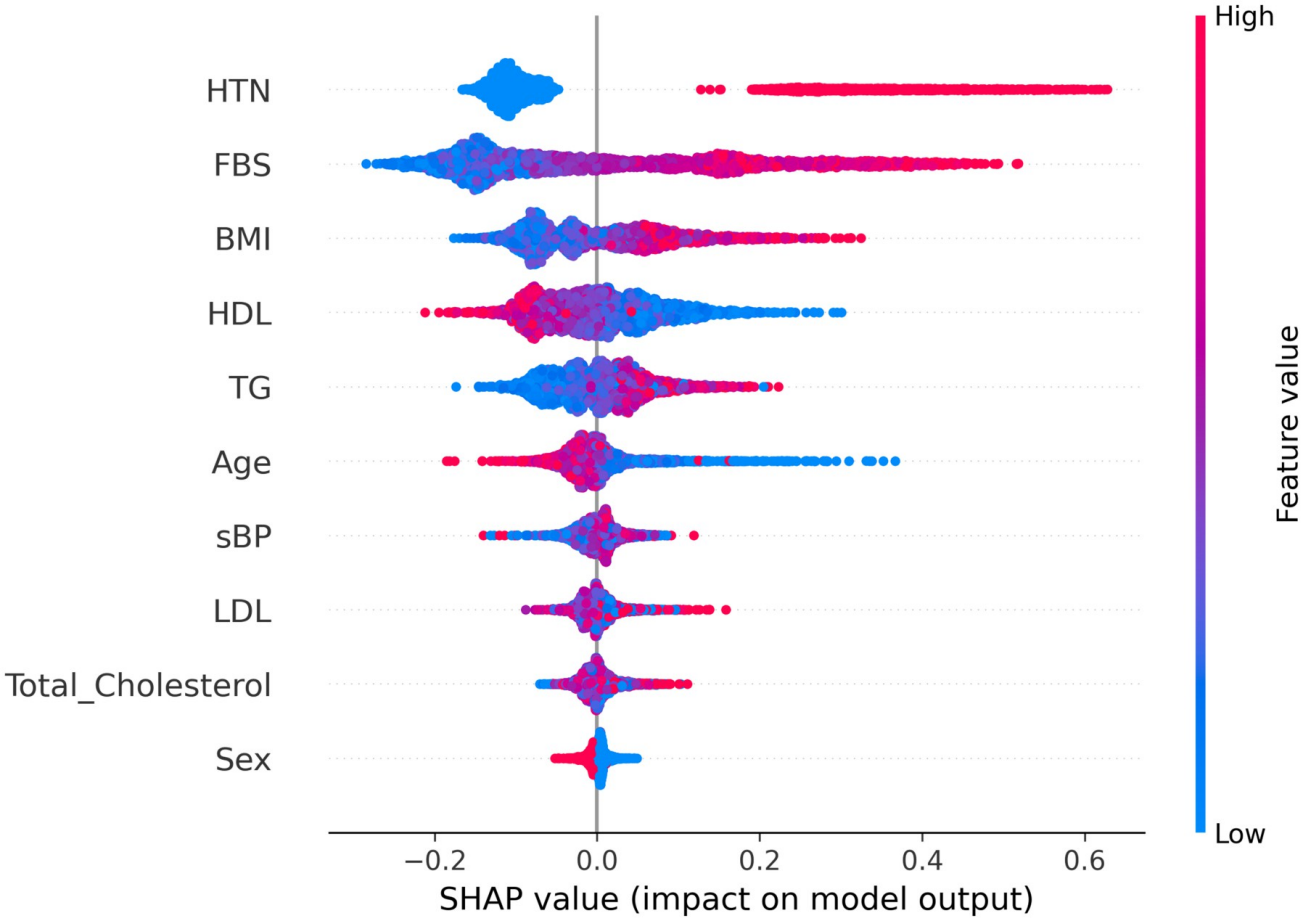
SHAP summary plot. Each point in the plot represents the SHAP value for a feature in an individual record of the dataset. The color represents the feature value from high (red) to low (blue). Features are ordered by importance.

Fig 4 represents individual explanations for the prediction of F1 (left panel) and F2 (right panel) in terms of SHAP values. This waterfall representation shows the positive or negative contribution of each feature in determining the output. Features are ordered according to their relevance, from the most relevant (top) to the least relevant (bottom). The SHAP analysis of patient F1 shows that the majority of the features have a positive contribution to the final prediction probability (*highT2DM* class), with the presence of hypertension being the most important. The high value of FBS is also crucial for the prediction, whereas BMI contributes in a slightly negative way as it lowers the output probability. Similarly, when considering patient F2, most of the features have a positive contribution towards the probability of *highT2DM* classification. In particular, the most relevant features are presence of hypertension, BMI, FBS and TG. SHAP values related to different individual explanations can be combined to get a comprehensive view of the features contribution over the whole set of data, as shown in Fig 5. This plot provides indications about the relationship between feature values and model output. Specifically, a higher probability to be in the *highT2DM* class is associated with high FBS, BMI, TG, LDL and Total Cholesterol, low HDL, and the presence of hypertension. The marginal contribution of Sex is almost negligible since the absolute SHAP value is always lower than 0.05.

By using DiCE as counterfactuals generation technique on the same set of 734 factuals, only 454 counterfactuals were found (availability = 62%). A not negligible portion of the factuals (i.e., about 20%) were misclassified by the Random Forest algorithm, therefore yielding to invalid counterfactuals. The discriminative power was high (average accuracy = 94%), whereas the similarity was equal, on average, to 16.2% of the maximum distance in the standardized features space (C.I.: 0%—32%).

## Discussion

The field of XAI has grown steadily in recent years, including applications in disease characterization and prediction. However, there is still lack of transparent approaches to generate personalized recommendations for T2DM prevention. This study addresses this research gap by introducing a new approach based on counterfactual explanations to quantify, in a personalized way, the minimum viable changes needed to improve a patient’s health status.

In this article, a TC-SVDD was trained and tested on a set of routinely collected biomarkers representing patients at high and low risk of developing T2DM, achieving a specificity of 91% and a sensitivity of 60%. It should be noted that, in this study, we focused on maximizing the ability to characterize the region where to identify counterfactuals (i.e., specificity and negative predictive value of the *lowT2DM* region) rather than on maximizing sensitivity as the class of the factuals was known by definition. Sensitivity may be increased in future studies by considering larger datasets and more specific biomarkers in addition to the primary care measures here used. The observed classification performance is in line with that of other predictive methods for T2DM available from the literature (i.e., sensitivity = 81%, 95% C.I.: 67%-90%; specificity = 82%, 95% C.I.: 74%-88% [3]). However, existing methods are typically able to provide general global recommendations that are not tailored to the individual patient’s characteristics, whereas the proposed approach defines personalized recommendations aimed at lowering the individual risk of developing T2DM on an individual basis.

### Counterfactuals generation and analysis

As described in the diagram shown in Fig 1, two different versions of the TC-SVDD were implemented. The canonical TC-SVDD provides very good results in defining regions that accurately enclose data points [17, 23, 27], whereas the *TC* − *SVDD*_*red*_ enables identification of more conservative minimum viable changes to lower the risk of developing T2DM because it identifies a smaller, but more reliable, target region for the *lowT2DM* class (FNR = 9% on the test set).

The distributions of the biomarkers changes suggested by the two versions of TC-SVDD have been investigated in four groups of patients, subdivided based on sex (male/female) and diagnosis of HTN (presence/absence) (Fig 2). Results have shown that *TC* − *SVDD*_*red*_ allows for more noticeable changes for almost all biomarkers (including for example sBP and BMI, as shown in Fig 2), for three out of four patient groups (i.e., F_HTN, M_HTN, and M_noHTN). More pronounced differences are observed when considering hypertensive patients (both females and males) with respect to non-hypertensives. Hence, counterfactuals generated from *S*_2_*red*_ turn out to be potentially more viable for generating possible recommendations than those generated from *S*2. For example, to lower the risk of developing T2DM, patient F1 shown in Fig 1 should achieve lower FBS and sBP values with both canonical TC-SVDD and *TC* − *SVDD*_*red*_, but the changes targeted by the latter are greater and presumably move the patient in a state that is further away from developing the disease compared to those targeted by the classical version of the classifier. Similar considerations can be made for patient F2, as shown in Fig 1.

A deeper analysis of counterfactuals generated using *TC* − *SVDD*_*red*_ showed that, in general, patients of all the four groups are, on average, suggested to reduce FBS, sBP and TG and increase HDL by an amount substantially different than 0 (Table 2). These changes relate to a global improvement in biomarker values. The demand to lower TG and increase HDL may coincide with the fact that a large number of T2DM patients suffer from dyslipidemia (abnormalities in lipid levels), notably characterized by elevated TG and LDL and reduced HDL, that may occur before the onset of the disease [25]. Moreover, the treatment of this condition may reduce the risk of developing cardiovascular disease which is the main cause of mortality in patients with T2DM [26]. Moreover, hypertensive patients are generally required to change their biomarkers to a greater extent than non-hypertensive patients. Specifically, as expected, *HTN* patients are asked to reduce their sBP values by a greater amount (i.e., median changes of about 5 mmHg for female patients and 7.5 mmHg for male patients) with respect to *noHTN* patients. In addition, also a greater reduction in FBS (i.e., median changes of about 1.25 mmol/L for female patients and 1.31 mmol/L for males patients) is required for *HTN* patients with respect to *noHTN* ones, suggesting a longitudinal connection between blood pressure values and T2DM [37] and highlighting the importance of keeping both blood pressure and blood glucose under control in order to reduce T2DM risk [1]. Considering the female groups, greater reductions in BMI and TG values were also found in hypertensives vs non-hypertensives patients. A possible future application of the proposed methodology may be the following: a patient goes to the primary care physician and all the necessary biomarkers are routinely measured and recorded into the EMR. The set of input features is fed to the previously trained *TC* − *SVDD*_*red*_. If the patient is classified in the *highT2DM* region, its counterfactual is generated and the doctor, based on medical expertise, may recommend a specific, individualized prevention strategy to target the proposed target values and reduce the T2DM risk. Several encounters may be scheduled to monitor the evolution of patient’s biomarkers in time until the patient is finally and effctively classified as *lowT2DM* by the *TC* − *SVDD*_*red*_.

One of the advantages of the proposed approach compared to global prediction models is that the counterfactual generation method takes into account all the controllable features for each real patient whereas global rule-sets derived from classic fully interpretable ML algorithms may involve only a subset of features, i.e. those that are representative of a sufficiently large part of the population, to limit overfitting. For example, a hypothetical rule-based model may classify patients in the *highT2DM* class if their age and blood pressure are above certain cutoffs. However, there may be specific patients with high age and high blood pressure that actually belong to the *lowT2DM* class because of the values of other features that are not taken into account by the main decision rules determined by the algorithm. Thus, the personalized approach presented in this study may provide a higher degree of flexibility and personalization as it allows us to identify (and therefore act) on specific features that are relevant for the individual case and to potentially apply tailored interventions.

When implementing new medical decision support methods, continuous interaction with the end users i.e., clinicians, is of paramount importance. For this reason, to gain a deeper insight into the feasibility and applicability of the proposed method, feedback from experts was collected in the form of an online survey.

To gather information regarding how medical experts evaluate the risk of T2DM given a pretty restricted set of routinely collected biomarkers, a *risk assessment* section was introduced in the survey. In this section, examples of subjects with the corresponding biomarkers were proposed, without making explicit whether or not these subjects will develop T2DM in one year (i.e., their “real” output class). Two of these examples are reported in Table 3 and briefly discussed below.

Surprisingly, EX1 was rated by the interviewed experts as having minor risk of developing T2DM with a pretty high confidence level despite having FBS higher than prediabetes threshold. Actually, EX1 is an example of instance belonging to *highT2DM* class. Presumably, this low rating is due to two main reasons. First, BMI is not too high and there may be lack of meaningful additional information like A1c, family history and exercise level. Second, assessing clinical risk is a task characterized by some degree of uncertainty due to the fact that there is a natural overlap between classes and *lowT2DM* and *highT2DM* patients cannot always be perfectly distinguished, especially when considering a limited set of features.

EX2 was coherently evaluated by clinicians based on what was stated for EX1. Indeed, EX2 is younger and his FBS is lower with respect to EX1 whereas BMI and Total Cholesterol values are comparable between the two patients and, because of this, EX2 risk was rated as lower or equal, compared with EX1, by 6 of 7 clinicians.

More generally, when experts were asked to assess the risk of patients, some variability in responses was observed. The heterogeneity characterizing the evaluations is also evident in the remaining survey responses, which are not explicitly reported in the “Results” section.

General questions regarding prior experience with AI and its perceived importance in the medical field were also included in the survey. As one might expect, the surveyed clinicians foresee that AI techniques will play a not negligible role in medicine in the upcoming years because of their many possible applications in supporting diagnosis, prognosis, prevention and management of diseases. However, the ability of these techniques to provide quantitative explanations is not enough to make the decision support system fully trustable. In this regard, several other requirements must be considered to implement Trustworthy AI solutions (e.g., [13, 14]) and decrease the skepticism that stakeholders (i.e., clinicians and patients) may have toward systems that can make decisions on their own.

To evaluate examples of target values derived from minimum viable changes in biomarkers, factual-counterfactual pairs were also included in the survey. Two out of five examples proposed to the experts are reported in the Results section (Table 4). All the experts agreed with the target values proposed to reduce the T2DM risk of patient F1 as the target goals were considered reasonable. In particular, lowering FBS was considered as a very important mechanism. The treatments proposed to achieve these goals included moderate healthy diet and regular exercise. In some cases, however, lowering T2DM risk may not be the priority for the clinician when looking at the biomarkers. For example, a very high LDL value may suggest high cardiovascular risk for the patient under examination. In this case, it could be more important to act on LDL reduction with respect to other features more associated with T2DM (e.g., FBS), as the presence of cardiovascular risk may lead to potential life-critical situations (e.g., miocardial infarction or stroke). Hence, future studies may propose a set of different counterfactuals for each patient generated according to different categories of risk.

The surveyed experts were in general agreement also with the values proposed to lower the risk of patient F2, since the targets to be achieved point towards the right direction with regard to risk reduction (i.e., lower FBS, sBP and BMI). However, in this case the respondents were particularly skeptical with concern to the BMI value. In fact, patient F2, who is severely obese according his BMI value (i.e., class 3 obesity), is asked for a reduction of 4*kg*/*m*^2^ which is judged too small and therefore ineffective in reducing T2DM risk. It is important to note that the proposed reduction still involves a 9% change in BMI, and furthermore, what we are aiming for is to find minimum viable changes that can be implemented in a relatively short time and not drastic changes. Nevertheless, it will be crucial in the future to introduce medical guidelines in the generation of counterfactuals, to be followed (when feasible) to achieve clinically sufficient changes.

When experts were asked to select the most significant biomarkers for their assessments, BMI and FBS were found to be the most relevant. Notably, FBS was not chosen by all respondents perhaps because it is subject to more day to day fluctuations than BMI, which is a well-known primary risk factor for T2DM and was considered more stable. In addition, FBS value might be more affected by external factors with respect to other correlated diagnostic markers like for example glycosylated hemoglobin (HbA1c). It should be emphasized that the experts responded based on the assessments proposed in the survey, so the obtained result is specific and may depend on the provided examples.

The majority of the respondents evaluated the proposed counterfactuals as realistic (4) and/or consistent (2). Skepticism is mostly due to the fact that the proposed target values may not be applicable to everyone. Indeed, patients with restricted mobility may not be able to exercise to achieve the necessary targets. Moreover, also patients stuck on a particular diet, may not be able to achieve the clinical goal. Metformin may be prescribed for T2DM prevention to lower FBS level and therefore contrast hyperglycemia by targeting the proposed values when lifestyle changes are not considered sufficient, however this medicine is not suitable for all people. Indeed, its prescription is not advised when the patient has liver or kidney diseases or if the patient has heart failure. Therefore, we need to have access to more information about the patients and eventually define a set of patients for which this kind of AI-supported strategy is applicable. This may lead to possible ethical biases in contrast with EU requirements for trustworthy AI (e.g., *Diversity, non-discrimination and fairness* requirement [13]).

For this reason, it should not be the automated medical decision support system to choose if the method is applicable but it is up to the clinicians employing the tool to choose whether or not to apply the proposed strategy based on their own experience. In any case, the patients keep the right to decide if they wish to proceed with the proposed prevention strategy.

We have compared the proposed method with different local XAI techniques. SHAP is a very useful technique to study the effect of specific feature values in predicting a certain output. As such, it may be used to identify relevant features for specific patients and indicate the desired direction for change in controllable characteristics (e.g., decrease in FBS in F1, decrease in BMI in F2) toward a reduced risk of developing T2DM. However, the SHAP technique is not able to directly provide quantitative personalized recommendations, in the form of target values, as counterfactuals do. For example, looking at Fig 4 we can say that patient F1 should mainly lower her FBS value and cure hypertension to lower the risk of having T2DM. Patient F2, in addition to cure hypertension and lower FBS of a smaller amount with respect to F1, should also lower BMI and TG. Although these suggestions are helpful, the generation of counterfactuals allows us to make a further step and define target biomarkers values that may be used as a basis to make personalized therapeutic recommendations. Comparative analysis of our method with respect to DiCE using the same set of 734 *highT2DM* patients showed that DiCE was worse in terms of availability of counterfactuals (i.e., 62% vs 99%) and in terms of similarity, as the average distance between factuals and counterfactuals was greater. The discriminative power was 94% in both cases, proving the ability of both methods to distinguish counterfactuals, that are ‘virtual’ *lowT2DM* patients, from real *highT2DM* patients. Based on these analyses, we can conclude that our method is more suitable for the purpose of generating minimum feasible changes.

### Limitations

This study presents some limitations. The first one relates to the data sample. In particular, the dataset considered includes a limited set of common primary care biomarkers which reflects the characteristics of a specific population based in Canada and may not be generalizable to other populations. Another limitation of this study relates to the classification using TC-SVDD. The optimization of the TC-SVDD was focused on the characterization of region *S*_2_ based on retrospective data with known output labels. For this reason, specificity was maximized, at the expense of sensitivity, and factuals were selected among real *highT2DM* patients. However, since in future we will deal with prospective data related to patients with unknown risk, an accurate representation of *S*_1_ will be important to find patients with a predicted high risk of developing *T2DM* (i.e., factuals). Therefore, a trade-off between these performance metrics must be found. Another limitation concerns the formulation of the survey. To avoid possible sources of bias and to ensure that responses were not guided in any way, the respondents received minimal information with respect to the proposed method. Therefore, different experts may have interpreted the questions differently. In addition, the questionnaire did not specify the time frame in which patients should achieve the target values. Interviewed experts often referred to larger changes to obtain a substantial risk reduction, not realistically achievable with short-term treatments. Finally, in this preliminary study, only a reduced number of counterfactuals were included in the survey to limit the completion time and the expert survey did not include specific comparison of counterfactuals generated using the proposed method with respect to DiCE.

### Future developments

Starting from the previously discussed limitations, several potential research lines can be explored in future studies. First of all, to achieve a more precise characterization of patients and target biomarkers values, a larger set of input features should be considered, including family history, medications, level of exercise, diet, alcohol consumption, cardiovascular risk (e.g., Framingham score [38]), presence of comorbidities, and socio-economic status. Moreover, causal relationships between features should be investigated to asses causation beyond association. It will be also necessary to validate the proposed method on patients from different geographical areas using data from longitudinal datasets, like e.g., the English Longitudinal Study of Ageing (ELSA) [39]. Further research will target the optimization of the TC-SVDD through the choice of hyperparameters that can minimize unclassified points and improve prediction performance. Currently, there is no standard metric to evaluate counterfactuals from a computational perspective, although some measures have been proposed in the literature [15] and applied in this study. Future works may focus on the application and further development of these metrics to compare our method with other existing techniques for counterfactuals generation. As a further development, the structure of the survey could be refined to limit possible biases, for instance by introducing a set of examples to help respondents familiarize themselves with the questions and by specifying more clearly that the method focuses on minimum viable changes. In the next studies, it will be also necessary to focus on a more extensive human-based evaluation. Moreover, the risk reduction obtained by applying counterfactuals should be evaluated also with respect to common clinical risk estimators such as the Canadian Diabetes Risk Questionnaire (cANRISK) [40]. Finally, future developments should take into account continuous interaction with a team of clinical experts to develop and validate a platform able to generate realistic personalized strategies that aim at lowering T2DM risk by targeting specific values.

## Conclusion

To our knowledge, this is the first study in which an XAI framework based on counterfactual explanations is specifically applied to reduce the risk of a chronic disease, based on personalized minimum viable recommendations. As an added value, the methodology here introduced has been compared to other solutions for the generation of local explanations and preliminarily evaluated by experienced clinicians. Results showed that the proposed method performed better than an alternative method for counterfactuals generation (i.e., DiCE) in terms of availability and similarity of counterfactuals. Moreover, changes in biomarkers recommended by the proposed method were consistent with local XAI analysis using SHAP and with medical knowledge, as suggested by the expert assessment and related literature. In principle, the proposed approach is able to define regions with various levels of confidence associated with different FNR cut-off thresholds, that can be easily controlled by changing the radius of the *S*_2_ region. In future, it may be useful to search for a path of step-wise individualized minimal changes in biomarkers’ values that may be readily achievable but at the same time enable progressive reduction of risk. This may be helpful to facilitate adherence to therapy which is an imperative factor for the success of any prevention strategy. Further research will focus on the examination of a larger set of modifiable risk factors in populations with different characteristics and on more extensive evaluation of the explanations from both methodological and clinical perspectives.

## Supporting information

S1 File Survey. Text of the online survey proposed to a team of clinical experts.
(PDF)
