# Supplementary material for: A novel method to derive personalized minimum viable recommendations for type 2 diabetes prevention based on counterfactual explanations
Source: PLoS One. 2022 Nov 17;17(11):e0272825. doi: 10.1371/journal.pone.0272825 (PMC9671330; doi:10.1371/journal.pone.0272825)
Supplement: S1 File — Text of the online survey proposed to a team of clinical experts. (PDF) [file pone.0272825.s001.pdf]

Questionnaire

Thank you for participating in this research study from Consiglio Nazionale delle Ricerche (CNR), Italy.

Artificial intelligence (AI) methods have received increasing attention in medical decision support in recent years. Specifically, eXplainable Artificial Intelligence (XAI) techniques allow the knowledge discovery process of AI models to be easily interpreted.
The purpose of this study is to evaluate a local XAI method for the generation of personalized Type 2 Diabetes prevention recommendations based on counterfactual explanations derived from routinely collected biomarkers from primary care Electronic Medical Records.

The questionnaire consists of four sections.
- In section 1 you will be presented with general questions about your practice and your opinion on AI.
- In section 2 you will be presented with examples of patient data. You will be asked to define if the patient is at risk of developing Type 2 diabetes in 1-year and to give a short justification of your response.
- In section 3 you will be presented with examples of personalized prevention strategies, specifically AI-estimated minimum corrections of biomarker values to lower the individual risk of developing Type 2 diabetes in 1 year. You will then be asked to indicate how much you agree with the proposed strategies.
- In section 4 you will be presented with some final questions to collect your feedback on the proposed methodology.

Participation to this survey is voluntary. You can withdraw anytime without penalty. Your confidentiality will be protected, to the extent permitted by applicable laws. We will do this by ensuring the data is anonymous.
If you have any questions about the research, please contact Marta Lenatti at marta.lenatti@ieiit.cnr.it.

* Obbligatoria

Your general opinion on AI in medical decision support systems

1. What is your medical specialty? *

◯ Diabetology

◯ Endocrinology

◯ Primary care

◯ Other

2. You answered 'Other' to the previous question, please tell us which is your medical specialty: *

[ ]

3. Do you have any knowledge/experience with artificial intelligence? *

- (1) No knowledge
- (2) Minimal knowledge
- (3) Basic knowledge
- (4) Adequate knowledge
- (5) Superior knowledge

4. On a scale from 1 to 5, how do you rate the impact Artificial Intelligence will have on medical decisions in the coming years? *

- (1) Insignificant
- (2) Minor
- (3) Moderate
- (4) Major
- (5) Important
- I don't know

5. How much do you agree with the following statement?

"An AI-based medical decision support system able to provide quantitative explanations may be considered fully trustworthy." *

- (1) Strongly disagree
- (2) Moderately disagree
- (3) Neither agree nor disagree
- (4) Moderately agree
- (5) Strongly agree

Assessment of the risk of developing Type 2 diabetes

6. A fe*male* patient is characterized by the following set of biomarkers:
Age=84 years; sBP= 126.5 mmHg; BMI= 27.0 kg/m^2 ; LDL= 2.6 mmol/L; HDL= 2.1 mmol/L; TG=1.3 mmol/L; FBS=6 mmol/L, Total Cholesterol= 4.7 mmol/L, no hypertension.
Given these values, how do you estimate the subject's risk of developing Type 2 diabetes within 1 year? *

- ◯ (1) No risk (remote?)
- ◯ (2) Minor risk (unlikely?)
- ◯ (3) Moderate risk
- ◯ (4) Major risk
- ◯ (5) Severe risk

7. How confident are you that your previous statement is correct? *

- ◯ (1) Not confident at all
- ◯ (2) Slightly confident
- ◯ (3) Somewhat confident
- ◯ (4) Fairly confident
- ◯ (5) Completely confident

8. You answered 'Not confident at all' to the previous question, why? *

9. A *male* patient is characterized by the following set of biomarkers:
Age=56 years; sBP= 124 mmHg; BMI= 27.40 kg/m^2 ; LDL= 3.2 mmol/L; HDL= 1.1 mmol/L; TG=0.9 mmol/L; FBS=4.7 mmol/L, Total Cholesterol= 4.8 mmol/L, no hypertension. Given these values, how do you estimate the subject's risk of developing Type 2 diabetes within 1 year? *

- ◯ (1) No risk (remote?)
- ◯ (2) Minor risk (unlikely?)
- ◯ (3) Moderate risk
- ◯ (4) Major risk
- ◯ (5) Severe risk

10. How confident are you that your previous statement is correct? *

- (1) Not confident at all
- (2) slightly confident
- (3) Somewhat confident
- (4) Fairly confident
- (5) Completely confident

11. You answered 'Not confident at all' to the previous question, why? *

12. A fe*male* patient is characterized by the following set of biomarkers:
Age=47 years; sBP= 102 mmHg; BMI= 21.64 kg/m^2 ; LDL= 2.87 mmol/L; HDL= 1.5 mmol/L; TG=0.4 mmol/L; FBS=4.7 mmol/L, Total Cholesterol= 4.55 mmol/L, no hypertension.
Given these values, how do you estimate the subject's risk of developing Type 2 diabetes within 1 year? *

- (1) No risk (remote?)
- (2) Minor risk (unlikely?)
- (3) Moderate risk
- (4) Major risk
- (5) Severe risk

13. How confident are you that your previous statement is correct? *

- (1) Not confident at all
- (2) slightly confident
- (3) Somewhat confident
- (4) Fairly confident
- (5) Completely confident

14. You answered 'Not confident at all' to the previous question, why? *

15. A *male* patient is characterized by the following set of biomarkers:
Age=68 years; sBP= 115 mmHg; BMI= 25.75 kg/m^2 ; LDL= 3.2 mmol/L; HDL= 1.1 mmol/L; TG=1.6 mmol/L; FBS=5.5 mmol/L, Total Cholesterol= 4.92 mmol/L, no hypertension.
Given these values, how do you estimate the subject's risk of developing Type 2 diabetes within 1 year? *

- (1) No risk (remote?)
- (2) Minor risk (unlikely?)
- (3) Moderate risk
- (4) Major risk
- (5) Severe risk

16. How confident are you that your previous statement is correct? *

- (1) Not confident at all
- (2) slightly confident
- (3) Somewhat confident
- (4) Fairly confident
- (5) Completely confident

17. You answered 'Not confident at all' to the previous question, why? *

18. A fe*male* patient is characterized by the following set of biomarkers:
Age=48 years; sBP= 133 mmHg; BMI= 32.5 kg/m^2 ; LDL= 2.7 mmol/L; HDL= 0.8 mmol/L; TG=1.0 mmol/L; FBS=4.3 mmol/L, Total Cholesterol= 4.7 mmol/L, no hypertension.
Given these values, how do you estimate the subject's risk of developing Type 2 diabetes within 1 year? *

- (1) No risk (remote?)
- (2) Minor risk (unlikely?)
- (3) Moderate risk
- (4) Major risk
- (5) Severe risk

19. How confident are you that your previous statement is correct? *

- ◯ (1) Not confident at all
- ◯ (2) slightly confident
- ◯ (3) Somewhat confident
- ◯ (4) Fairly confident
- ◯ (5) Completely confident

20. You answered 'Not confident at all' to the previous question, why? *

21. A *female* patient with the following biomarkers is at high risk of developing Type 2 diabetes (1 year estimation):
Age=46 years; sBP= 132 mmHg; BMI= 35.60 kg/m^2 ; LDL= 3.5 mmol/L; HDL= 0.7 mmol/L; TG=2.4 mmol/L; FBS=4.6 mmol/L, Total Cholesterol= 5.37 mmol/L, no hypertension.
The algorithm proposes to lower the risk of developing diabets by suggesting a strategy that targets the following values:
sBP= 133mmHg; BMI= 33.60 kg/m^2 ; LDL= 2.9 mmol/L; HDL= 1.6 mmol/L; TG=2.6 mmol/L; FBS=4.5 mmol/L, Total Cholesterol= 4.7 mmol/L.
How much do you agree with the algorithm proposal? *

- (1) Strongly disagree
- (2) Moderately disagree
- (3) Neither agree nor disagree
- (4) Moderately agree
- (5) Strongly agree

22. Why?

23. What treatment would you recommend to help the patient make the changes required to achieve the suggested targets?

24. A *female* patient with the following biomarkers is at high risk of developing Type 2 diabetes (1 year estimation):
Age=63 years; sBP= 133 mmHg; BMI= 28.70 kg/m^2 ; LDL= 3.1 mmol/L; HDL= 1.1 mmol/L; TG=1.5 mmol/L; FBS=6.2 mmol/L, Total Cholesterol= 4.9 mmol/L, hypertension.
The algorithm proposes to lower the risk of developing diabets by suggesting a strategy that targets the following values:
sBP= 114 mmHg; BMI= 25 kg/m^2 ; LDL= 3.0 mmol/L; HDL= 0.8 mmol/L; TG=0.4 mmol/L; FBS=4.5 mmol/L, Total Cholesterol= 3.8 mmol/L. How much do you agree with the algorithm proposal? *

- (1) Strongly disagree
- (2) Moderately disagree
- (3) Neither agree nor disagree
- (4) Moderately agree
- (5) Strongly agree

25. Why?

26. What treatment would you recommend to help the patient make the changes required to achieve the suggested targets?

27. A *male* patient with the following biomarkers is at high risk of developing Type 2 diabetes (1 year estimation):
Age=55 years; sBP= 157 mmHg; BMI= 44.10 kg/m^2 ; LDL= 3.6 mmol/L; HDL= 1.2 mmol/L; TG=2.3 mmol/L; FBS=5.8 mmol/L, Total Cholesterol= 5.9 mmol/L, hypertension.
The algorithm proposes to lower the risk of developing diabets by suggesting a strategy that targets the following values:
sBP= 134 mmHg; BMI= 40 kg/m^2 ; LDL= 3.0 mmol/L; HDL= 1.2 mmol/L; TG=2.0 mmol/L; FBS=5.0 mmol/L, Total Cholesterol= 6.2 mmol/L. How much do you agree with the algorithm proposal? *

- (1) Strongly disagree
- (2) Moderately disagree
- (3) Neither agree nor disagree
- (4) Moderately agree
- (5) Strongly agree

28. Why?

29. What treatment would you recommend to help the patient make the changes required to achieve the suggested targets?

30. A *male* patient with the following biomarkers is at high risk of developing Type 2 diabetes (1 year estimation):
Age=70 years; sBP= 127 mmHg; BMI= 26.79 kg/m^2 ; LDL= 5.2 mmol/L; HDL= 1.3 mmol/L; TG=0.8 mmol/L; FBS=5.2 mmol/L, Total Cholesterol= 6.8 mmol/L, no hypertension.
The algorithm proposes to lower the risk of developing diabets by suggesting a strategy that targets the following values:
sBP= 122 mmHg; BMI= 25.25 kg/m^2 ; LDL= 3.1 mmol/L; HDL= 2.3 mmol/L; TG=0.6 mmol/L; FBS=4.1 mmol/L, Total Cholesterol= 5.4 mmol/L. How much do you agree with the algorithm proposal? *

- (1) Strongly disagree
- (2) Moderately disagree
- (3) Neither agree nor disagree
- (4) Moderately agree
- (5) Strongly agree

31. Why?

32. What treatment would you recommend to help the patient make the changes required to achieve the suggested targets?

Overall feedback

33. Do you think that overall the statements generated by the proposed method are realistic and/or consistent? *

- ◯ neither realistic nor consistent
- ◯ realistic but not consistent
- ◯ consistent but not realistic
- ◯ both realistic and consistent

34. Why?

35. Considering your responses to the questions in Section 2 and 3, which biomarkers have been more relevant in your assessments of the risk of developing Type 2 diabetes? *

Check all that apply

- ☐ BMI
- ☐ sBP
- ☐ FBS
- ☐ HDL
- ☐ LDL
- ☐ TG
- ☐ Total Cholesterol
- ☐ Diagnosis of Hypertension

36. Do you think the proposed Artificial Intelligence methodology can be useful in formulating personalized prevention strategies? *

- ◯ (1) Not at all useful
- ◯ (2) Not very useful
- ◯ (3) Neutral
- ◯ (4) Quite useful
- ◯ (5) Extremely useful

37. Please, provide any additional feedback:

Questo contenuto non è stato creato né approvato da Microsoft. I dati che invii verranno recapitati al proprietario del modulo.

Microsoft Forms
